# Long Tract of Untranslated CAG Repeats Is Deleterious in Transgenic Mice

**DOI:** 10.1371/journal.pone.0016417

**Published:** 2011-01-21

**Authors:** Ren-Jun Hsu, Kuang-Ming Hsiao, Min-Jon Lin, Chui-Yen Li, Li-Chun Wang, Luen-Kui Chen, Huichin Pan

**Affiliations:** 1 Institute of Medicine, Chung Shan Medical University, Taichung, Taiwan, Republic of China; 2 Department of Life Science, National Chung Cheng University, Chia-Yi, Taiwan, Republic of China; 3 Department of Biomedical Sciences, Chung Shan Medical University, Taichung, Taiwan, Republic of China; 4 Department of Medical Research, Chung Shan Medical University Hospital, Taichung, Taiwan, Republic of China; University of Pennsylvannia, United States

## Abstract

The most frequent trinucleotide repeat found in human disorders is the CAG sequence. Expansion of CAG repeats is mostly found in coding regions and is thought to cause diseases through a protein mechanism. Recently, expanded CAG repeats were shown to induce toxicity at the RNA level in *Drosophila* and *C. elegans*. These findings raise the possibility that CAG repeats may trigger RNA-mediated pathogenesis in mammals. Here, we demonstrate that transgenic mice expressing *EGFP* transcripts with long CAG repeats in the 3′ untranslated region develop pathogenic features. Expression of the transgene was directed to the muscle in order to compare the resulting phenotype to that caused by the CUG expansion, as occurs in myotonic dystrophy. Transgenic mice expressing 200, but not those expressing 0 or 23 CAG repeats, showed alterations in muscle morphology, histochemistry and electrophysiology, as well as abnormal behavioral phenotypes. Expression of the expanded CAG repeats in testes resulted in reduced fertility due to defective sperm motility. The production of EGFP protein was significantly reduced by the 200 CAG repeats, and no polyglutamine-containing product was detected, which argues against a protein mechanism. Moreover, nuclear RNA foci were detected for the long CAG repeats. These data support the notion that expanded CAG repeat RNA can cause deleterious effects in mammals. They also suggest the possible involvement of an RNA mechanism in human diseases with long CAG repeats.

## Introduction

The expansion of unstable trinucleotide repeats underlies a number of human disorders, which can be grouped into two categories according to the location of the repeats. In the first category, dominantly inherited neurodegenerative disorders are triggered by the expansion of CAG repeats located in the coding region. Examples of disorders belonging to this group include Huntington's disease (HD) and spinocerebellar ataxias (SCAs) types 1, 2, 3, 6, 7, and 17. In the second category, the expansion of different repeats, including CGG, GAA, CTG, and CAG, occur within the non-coding or untranslated regions (UTR), leading to fragile X syndrome (FRAX), Friedreich's ataxia (FRDA), myotonic dystrophy type 1 (DM1), SCA8, and SCA12 [1], [2], [3], [4], [5].

Most CAG expansions are less than 150 repeats and are located in coding regions and translated into polyglutamine tracts within the corresponding protein. Despite that the expansions occur over a wide range of genetic loci, there are common features to these disorders, suggesting that they may share a similar pathogenic mechanism. Toxic gain of function involving formation of polyglutamine aggregates, protein misfolding and transcriptional dysregulation, has been shown to result in neuronal cell toxicity [1], [2], [6]. In addition, loss of neurotrophic support due to reduced protein activity caused by polyglutamine expansions may also contribute to the pathogenesis of neurodegeneration [7].

In contrast to the repeat expansions located within coding regions, expansions located outside of coding sequences are usually very large and do not alter the sequences of the affected proteins. Recent studies have revealed a role for RNA in the pathogenesis of the dominantly inherited non-coding repeat disorders [5], [8], [9]. The best-studied example of this type is DM1. DM1 is a multisystemic disorder characterized by skeletal muscle wasting and myotonia, cardiac conduction defects, insulin resistance and cataracts. It is caused by an expansion of CTG repeats in the 3′ UTR of the *DMPK* gene [10]. Studies in animal models have shown that haploinsufficiency of the DMPK protein contributes only partially to the DM1 phenotype [11], [12]. In contrast, mice expressing mRNA with long CUG repeats in the 3′ UTR of either *DMPK* or an unrelated transgene developed the major features of DM1 [13], [14], [15]. These findings, together with the discovery that expansion of CCTG repeats in a second locus (DM2, located in intron 1 of the *ZNF 9* gene) also leads to a clinical presentation that is strikingly similar to DM1 [16], indicate that the expanded repeats act trans-dominantly. The transcripts of expanded CUG/CCUG repeats form highly stable hairpin structures [17] and accumulate as foci in the nucleus [18], [19], [20]. The muscleblind-like (MBNL) proteins, which bind to double-stranded CUG/CCUG repeats *in vitro* [21], [22], have been shown to colocalize with these RNA foci in DM tissues [20], [22], [23]. A model has been proposed in which the sequestration of the MBNL proteins, along with the accompanying upregulation of CUG-binding proteins [24], [25], results in disruption of alternative splicing of genes that are misregulated in DM [26], [27], [28], leading to the multisystemic clinical features [3]. Consistent with this model, both *muscleblind* knockout mice and transgenic mice overexpressing CUG-BP1 displayed the pathological features and the splicing misregulation that are associated with DM [29], [30].

RNA-mediated pathogenesis is not limited to CUG repeats. For example, the CGG repeats in the Fragile X premutation range (60–200 repeats) cause a clinically distinct disorder called Fragile X tremor ataxia syndrome (FXTAS) [31]. Recent studies using a *Drosophila* model suggest that the CGG repeat-containing RNA is pathogenic [32], [33], [34]. This observation raises an interesting question as to whether other triplet expansions, such as CAG repeats, can have a pathogenic role at the RNA level. Indeed, not all disease-causing CAG repeats are in the coding region. For example, CAG repeats in SCA12 are located in the untranslated region. CAG repeats in the disease-related transcripts can form stable hairpin structures similar to those detected in DM1 [17], [35], [36], and RNA-binding proteins that specifically interact with CAG repeat sequences have been reported [37]. Furthermore, long CAG repeats formed RNA foci when introduced into cultured cells, and MBNL1 colocalized with these foci [38].

However, controversial results have been reported in *Drosophila* models of CAG expansions. In a study comparing the effects of expanded coding CAG/CAA repeats and untranslated CAG repeats, only constructs expressing polyglutamine at the protein level were shown to cause neurodegeneration [39], thus ruling out a role for CAG RNA as the pathogenic agent. On the other hand, modifier screening in the *Drosophila* models of SCA1 and SCA3 led to identification of genes that encode RNA-binding proteins [40] and the muscleblind protein [41], respectively, suggesting that alteration of RNA processing is relevant to SCA pathogenesis. Introduction of a SCA3 transgene containing CAG/CAA-interrupted codons (which encoded the identical amino acids) into flies dramatically mitigated the toxicity observed in flies expressing the pure CAG-encoded protein [41], which further supports the notion that RNA toxicity caused by CAG repeats participates in the neuronal degeneration in SCA3. Moreover, we have recently demonstrated that expanded CAG repeat RNA was toxic in *C. elegans* [42]. Our finding suggests that the pathogenic effect of CAG repeat RNA could be evolutionarily conserved.

To directly test the pathogenic role of CAG repeat RNA in a mammalian system, we generated transgenic mice expressing short and long CAG repeats in the 3′ UTR of the *enhanced green fluorescent protein* (*EGFP*) gene. Here, we present evidence showing that normal muscle physiology and sperm motility are impaired in the long CAG-expressing mice. These results suggest that RNA containing expanded CAG repeats can be deleterious to mammals as well as invertebrates.

## Results

### Generation of transgenic mice

**Figure 1:**
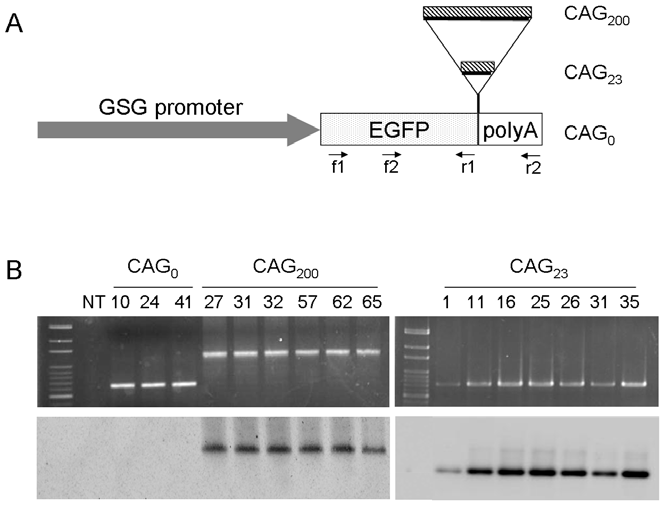
Generation of transgenic mice. (A) Diagram of the transgene constructs. The transgenes contain the gamma-sarcoglycan (GSG) promoter fused with the *EGFP* coding sequence and followed by SV40 polyadenylation signals (poly A). Insertion of 23 or 200 CAG repeats was made downstream of the EGFP stop codon and before the poly A sequence. Locations of primers (f1, f2, r1 and r2) used for PCR are marked. (B) PCR-based Southern blot analysis. Tail DNA from different founder animals (as indicated by the numbers) generated from the three constructs were PCR-amplified using primers f1 and r2, which generated fragments of 632 bp, 701 bp and 1,232 bp, from CAG_0_, CAG_23_ and CAG_200_ transgenes, respectively. Upper panels, ethidium bromide-stained agarose gels; lower panels, blots of PCR products hybridized with a CAG_10_ probe.

To determine if untranslated CAG repeat expansion causes pathogenic effects, we generated transgene constructs containing the *EGFP* gene with 0, 23 or 200 CAG repeats (designated as CAG_0_, CAG_23_ and CAG_200_, respectively) in the 3′ UTR (Fig. 1A). Transgene expression was directed to the skeletal muscle with the *gamma-sarcoglycan* (*gsg*) promoter [43] in order to compare the effects of CAG repeats to those caused by CTG repeats [13], [14]. Transgenic mice were initially screened by PCR and the results were subsequently confirmed by Southern blot analyses. The presence of CAG repeat sequences with the correct length in the transgenic mice was also checked by PCR-based Southern blot using a (CAG)_10_ oligomer as a probe (Fig. 1B). Three CAG_0_ (CAG_0_-10, -24 and -41), 7 CAG_23_ (CAG_23_-1, -11, -16, -25, -26,-31 and -35) and 6 CAG_200_ (CAG_200_-27, -31, -32, -57, -62 and -65) transgenic founder animals were generated and bred to establish independent lines. The transgenic animals used for subsequent analyses were randomly selected from the lines shown in Table S1.

### Transgene expression

**Figure 2:**
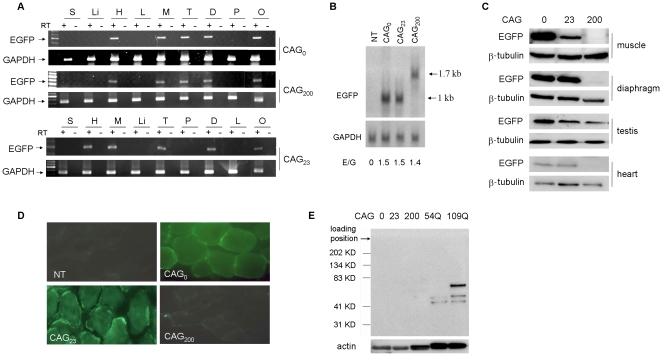
Transgene expression. (A) RT-PCR of RNA isolated from different adult tissues. Representative gels showing expression of *EGFP* in CAG_0_, CAG_23_ and CAG_200_ lines. Amplification of *GAPDH* was used as an internal control. +, with RT; −, without RT. S, skin; Li, liver; H, heart; L, lung; M, muscle (soleus); T, testis; D, diaphragm; P, pancreas; O, ovary. (B) Northern blot analysis. Representative blot showing skeletal muscle RNA from non-transgenic (NT), CAG_0_, CAG_23_ and CAG_200_ animals hybridized with an *EGFP* probe or a control *GAPDH* probe. Relative RNA expression levels (E/G, *EGFP* divided by *GAPDH*) are shown below. (C) EGFP protein expression in the transgenic lines determined by western blotting using an anti-EGFP antibody. H, heart; M, muscle; D, diaphragm. Expression of β-tubulin was used as a loading control. (D) EGFP fluorescence was observed using frozen sections from the soleus muscle with a fluorescence microscope. Reduced fluorescence in CAG_200_ muscle is consistent with the reduced protein expression shown in (C). (E) Detection of polyglutamine-containing protein with a mouse anti-polyglutamine monoclonal antibody (1C2). Protein extracts from the muscle of three mouse lines (50 µg each) and two cell lines transfected with expanded ataxin-3 proteins (54Q, 109Q) (12.5 µg each) were used. Arrowheads indicate the endogenous (45 Kd) and mutant ataxin-3 proteins with 54 or 109 CAG repeats. The position of the wells is indicated to verify the absence of insoluble 1C2-positive proteins. Actin expression was used as a control.

The expression of each transgene was first verified by RT-PCR analysis. The *EGFP* transcripts were present in heart, skeletal muscle, diaphragm, testis and ovary (Fig. 2A), consistent with the reported tissue specificity of the *gsg* promoter [43]. Independent lines of CAG_0_ (-10, -41), CAG_23_ (-16, -31, -35) and CAG_200_ (-32,-57, -62) were analyzed, and all lines exhibited similar patterns of tissue distribution. Northern blotting revealed that the length of the EGFP transcript in the CAG_200_ lines was approximately 600 bp longer than that in the CAG_0_ lines, as expected (Fig. 2B). In all analyzed lines, there was no noticeable difference in the levels of transcript expression. However, the EGFP protein levels were significantly reduced in the tissues of the analyzed CAG_200_ lines (Fig. 2C). This decreased protein expression was also reflected in the low GFP fluorescence intensity observed in CAG_200_ muscle sections using a fluorescence microscope (Fig. 2D). Thus, the expression of long CAG repeats in the 3′ UTR of the *EGFP* transcript resulted in decreased protein production.

To determine if cryptic translation leading to polyglutamine-containing protein product occurred in the transgenic lines, we performed western blotting using an anti-polyglutamine monoclonal antibody (1C2). No suspicious proteins with polyglutamine tracts were detected in tissue extracts from the transgenic lines (Fig. 2E); thus, the potential for polyglutamine protein contribution to the phenotypes described below was excluded.

### Histological analysis of muscle

**Figure 3:**
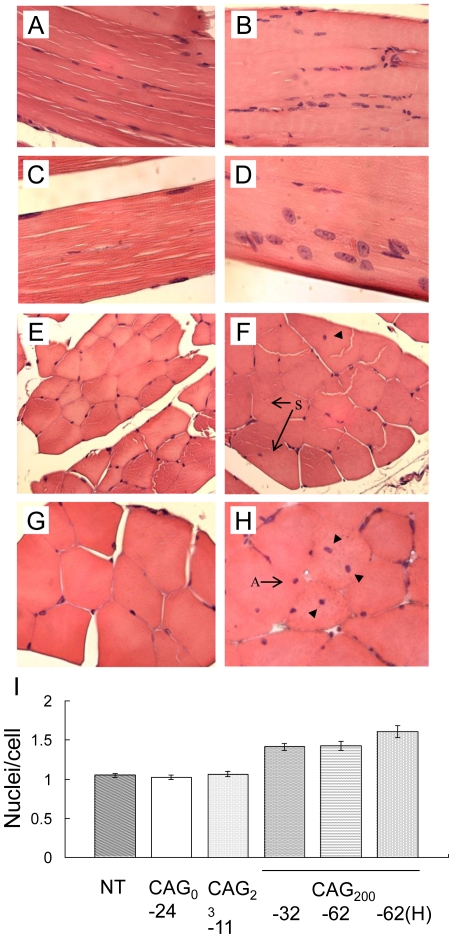
Muscle morphology. Hematoxylin and eosin-stained paraffin sections of soleus muscle oriented as longitudinal (A–D) or transverse sections (E–H) from 2-month-old non-transgenic (A, E), CAG_0_ (C), CAG_23_ (G), and CAG_200_ (B, D, F, H) animals. Note that the fiber diameters in CAG_200_ mice (B) are not as uniform as in the control (A) and that multiple rounded nuclei are observed (D). Some nuclei are located internally (arrowheads, F, H) instead of peripherally (C, E, G), and there are signs of split fibers (arrows in F) and angular fibers (arrow in H). (I) Quantification of nuclei (expressed as the nuclei-to-cell ratio). Nuclei and cells in 10 fields in each of four sections from each transgenic line (NT, CAG_0_-24, CAG_23_-11, CAG_200_-32, -62 and one homozygous mouse) were counted using 400× magnification; the average values are presented. *P<0.001. (A–B), 200×; (C–D), 400×; (E–F), 250×; (G–H), 400×.

Skeletal muscle from adult transgenic mice (Table S1) was prepared for histological examination of the muscle morphology. No signs of fibrosis, inflammation or regeneration in muscle fibers from any of the mice were observed. In non-transgenic (NT), CAG_0_ and CAG_23_ mice, the shape and size of the muscle fibers were homogeneous, and most nuclei were peripherally situated (Fig. 3A, C, E, G). In CAG_200_ mice, we observed muscle fibers of various sizes and shapes, increased intermyofibrillar connective tissue, split fibers and centronucleated fibers (Fig. 3B, D, F, H). In addition, the nuclei appeared larger, and the relative nucleus-to-fiber ratios for two CAG_200_ lines (1.41±0.04 and 1.42±0.06) were higher than those for NT and CAG_0_ mice (1.05±0.03 and 1.02±0.03, respectively; p<0.001) (Fig. 3I).

**Figure 4:**
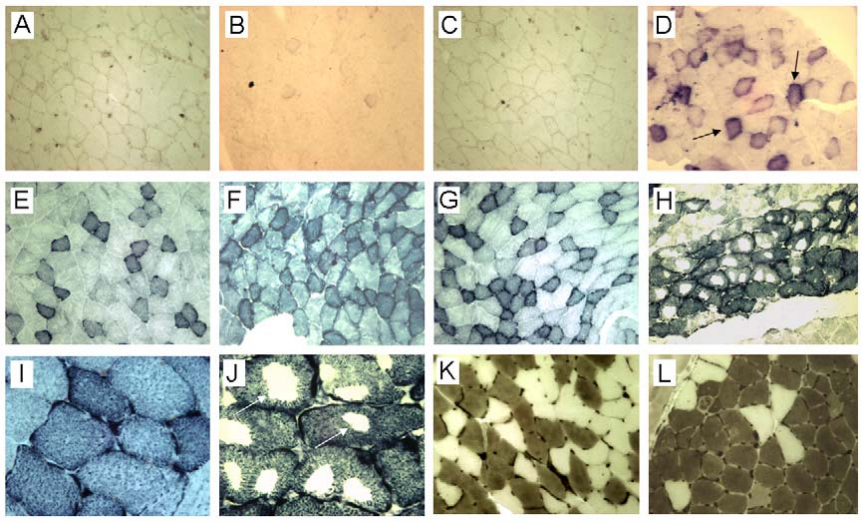
Histochemical analysis of muscle. Representative results showing frozen sections of the soleus muscle from 8-month-old non-transgenic (A, E, I), CAG_0_^-^24 (B, F), CAG_23_-11 (C, G, K) and CAG_200_-32 (D, H, J, L) animals. (A–D) Staining for succinate dehydrogenase (SDH). Note that normal fibers show very little reaction, whereas “ragged blue” fibers (arrows) are detected in lines CAG_200_-32 (D) and CAG_200_-62 (data not shown). (E–J) Staining for NADH-tetrazolium reductases. Note the altered fiber type grouping, moth-eaten patterns, and focal lack of intermyofibrillar network enzyme activity (H, J, arrows) in CAG_200_ mice compared to clear fiber type distinctions (E–G) and uniform lattices (I) in control animals. (K, L) Staining for ATPase activity at pH 4.3. Type I fibers are black and type II fibers are cream. Type I predominance is seen in CAG_200_ mice (L). A–H, 160×; I–J, 400×; K–L, 250×.

Histochemical staining for the oxidative reaction by succinate dehydrogenase (SDH) and for nicotinamide adenine dinucleotide reductase (NADHR) also revealed abnormal staining patterns in CAG_200_ mice. A modified SDH reaction using phenazine methylsulfate revealed the presence of fibers with heavy staining known as “ragged blue” fibers in sections from CAG_200_ lines (Fig. 4D). The heavy staining reflected a marked excess of mitochondria and was not seen in the muscle of control or CAG_23_ mice (Fig. 4A–C). The fibrillar network of NADHR reactivity is shown in Fig. 4E–J. The intermyofibrillar lattice pattern and subsarcolemmal pockets of NADHR reactivity mark the normal distribution of mitochondria (Fig. 4E, I). Both CAG_0_ and CAG_23_ mice showed a similar pattern to that observed in NT (Fig. 4F, G). In CAG_200_ mice, however, the network was irregular. Many fibers, both type I and type II, displayed an unusual “hollow” lattice pattern in which NADHR activity was lacking in one or more areas within the fibers (Fig. 4H, J). These “moth-eaten” fibers were not artifacts because they were reproducible in multiple mice from three CAG_200_ lines. Finally, staining for ATPase activity revealed that type I fibers were predominant and grouped in the soleus muscle of CAG_200_ mice (Fig. 4L). In contrast, the two fiber types appeared to be present in similar numbers and were randomly distributed in a similar muscle area in NT, CAG_0_ (data not shown) and CAG_23_ mice (Fig. 4K).

### Phenotype analysis

**Figure 5:**
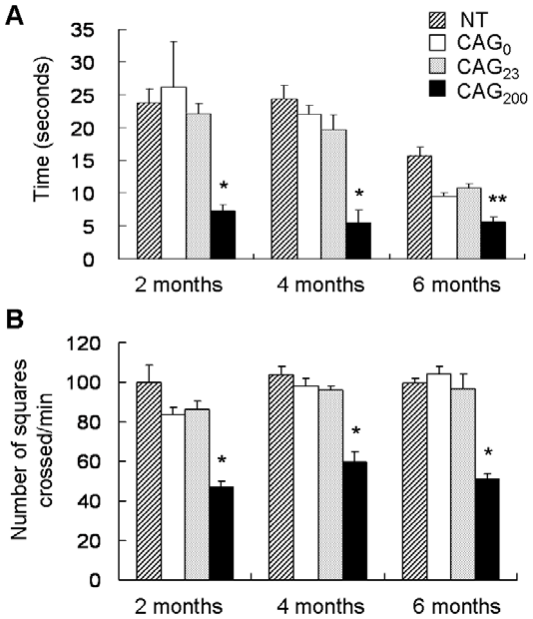
Phenotypic analysis of transgenic mice. (A) Grip strength test. Mice were suspended by the forelimbs on a narrow bar and the amount of time before falling was recorded. (B) Cage activity test. CAG_200_ lines displayed reduced locomotion activity as measured by a grid assay. All data represent the averages from 5 mice that were each tested 3 times (A and B). *, *P*<0.001; **, *P*<0.01 (compared to NT, CAG_0_, or CAG_23_; F test).

Mice expressing the CAG_200_ construct showed normal weight gain and did not have an overt phenotype. However, some of these mice occasionally displayed bizarre postures and had intermittent convulsions. To obtain a quantifiable phenotype assessment, we carried out a series of behavioral and functional analyses [44]. Results from two of these assays showed significant difference between control and CAG_200_ mice. When assayed by a locomotor activity test, the CAG_200_ mice showed reduced cage activity, as they crossed fewer squares per minute (Fig. 5A). In a narrow bar hang assay, these mice became fatigued and fell more quickly than control animals, suggesting that they had a muscle weakness phenotype (Fig. 5B).

In addition to the muscle phenotype, CAG_200_ mice also displayed reduced fertility. The mean litter size in CAG_200_ lines was similar to the CAG_0_ control mean value (7.6 pups per litter over 20 litters versus 8.4 pups per litter over 31 litters, respectively); however, breeding efficiency was greatly reduced in CAG_200_ lines. The time between new litters in a heterozygous CAG_200_-NT cross ranged from 6 to 8 weeks, compared to a range of 3 to 4 weeks in a normal or CAG_0_-NT cross. It took 9 to 12 weeks for a litter to be born from a mating between two heterozygous CAG_200_ mice, if any litter was born; approximately 30% of these matings resulted in no offspring at all.

### Sperm function and mitochondrial activity

**Figure 6:**
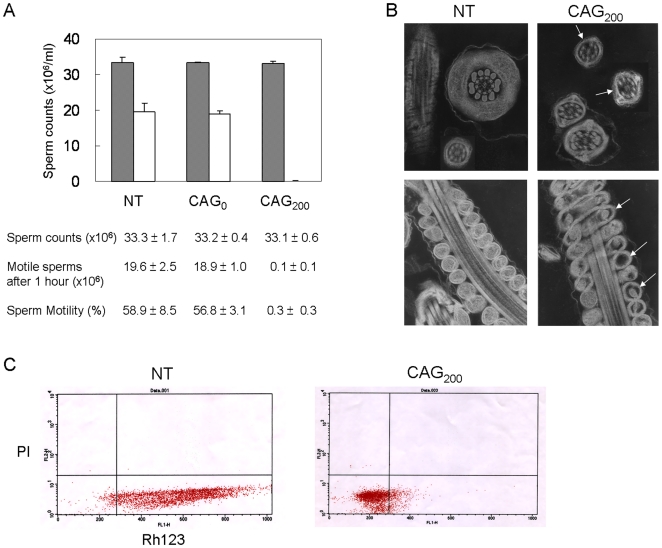
Sperm motility and mitochondrial function. (A) Counts of total sperm (gray) and motile sperm (white) after a 1-hr incubation at 37°C (*n* = 6 per group). The average counts are shown below the histogram. Percent sperm motility was determined by dividing the number of motile sperm by total sperm counts. (B) Structure of the sperm tails. Electron microscopy revealed structural defects in the microtubule arrangement of axonemes (loss of one outer doublet; upper right panel, arrows) and in the mitochondria along the midpiece (lower right panel, arrows) in some sperm tails of CAG_200_ mice. (C) Flow cytometric sorting of rhodamine (Rh123)- and propidium iodide (PI)-stained sperm cells. Horizontal scale, intensity of Rh123; vertical scale, intensity of PI. Note that most sperm cells from CAG_200_ males are gated with low PI and low Rh123 fluorescence, indicating that they are viable, but have low mitochondrial activity.

To understand the basis for reduced fertility in CAG_200_ mice, we first examined tissue sections from the testes, epididymis and ovaries. However, no significant differences were found in the size or histological features of these tissues between control and CAG_200_ mice (data not shown). Further analysis revealed that, although the sperm counts per epididymis were comparable, the motility of sperm from CAG_200_ males was greatly reduced to 0.3% over a 1-hour time frame (Fig. 6A) compared to 57–59% motility in CAG_0_ and NT males. When examined by electron microscopy, approximately two-thirds of the CAG_200_ sperm showed structural defects in their axonemes (Table S2), with loss of one outer doublet from the normal 9+2 microtubule arrangement (Fig. 6B, top panels). In addition, empty and abnormally shaped mitochondria were observed along the midpiece (Fig. 6B, lower panels).

To evaluate mitochondrial function, spermatozoa were stained with rhodamine123 (Rh123) and propidium iodide (PI) and subsequently sorted by flow cytometry. Rh123 specifically accumulates in the mitochondria and is recognized as an indicator of mitochondrial membrane potential (MMP), which has been shown to correlate positively with sperm motility [45], [46]. As shown in Fig. 6C, most of the sperm cells from control males had relatively high Rh123 fluorescence, whereas most sperm cells from CAG_200_ males had low Rh123 fluorescence. All of the spermatozoa had low PI fluorescence, indicating that they were viable. This analysis confirmed that sperm from CAG_200_ males had defective mitochondrial activity, which led to reduced sperm motility and fertility.

### Muscle electrophysiology

**Figure 7:**
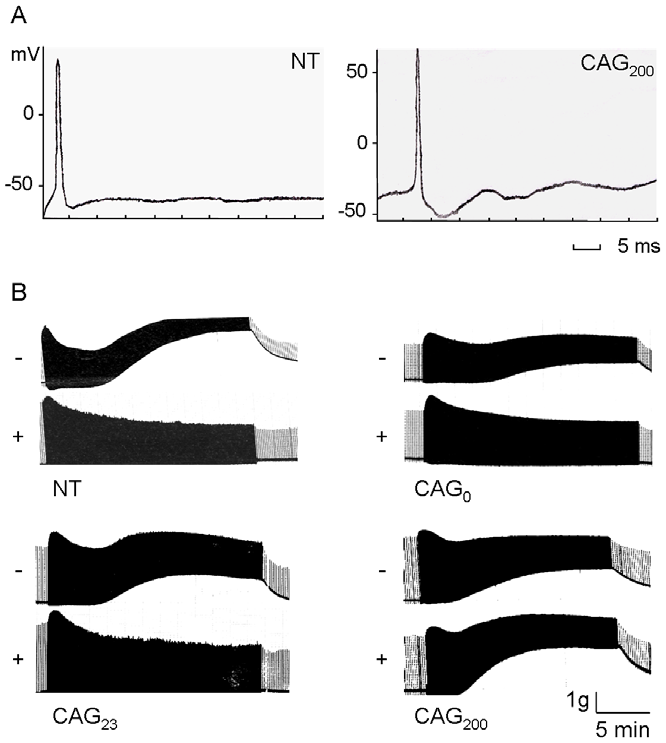
Electrophysiology of the muscle. (A) Action potential. A single action potential was triggered with a depolarizing current of 12 nA at 50-ms duration in NT and CAG_200_ mice. (B) Phrenic nerve-evoked contracture of isolated diaphragm. In the presence of glucose, significant contracture was induced by a 5-Hz stimulation for 20 min in CAG_200_, but not NT, CAG_0_ or CAG_23_ mice. As a control, all mice produced muscle contracture with a glucose-free Krebs solution using the same stimulation method. +, with glucose; −, without glucose.

Previous studies have shown that expression of untranslated CUG expansions in the skeletal muscle can result in repetitive electromyographic discharges [13], [14], [47]. To determine if there were defects in muscle membrane conductance in CAG_200_ mice, sharp microelectrode recordings were performed on excised diaphragm muscle fibers. A single action potential was triggered in NT or CAG_200_ fibers by applying a 12 nA current (Fig. 7A). No difference in the current threshold or latency was observed, indicating that muscle membrane conductance was not impaired by the CAG expansion.

Because the patterns of mitochondrial enzyme activity were altered in the muscle of CAG_200_ mice (Fig. 4), we next measured muscle contracture upon nerve-evoked stimulation. As shown in Fig. 7B, contracture of the isolated phrenic-nerve diaphragm was induced significantly by 5 Hz nerve-evoked stimulation for 20 min in CAG_200_ but not in NT, CAG_0_, or CAG_23_ mice (lower panels). Table 1 summarizes the contracture force detected in the different transgenic lines. In contrast, under metabolic stress conditions (glucose-free Krebs solution), contracture was induced in the diaphragms of all mice, including controls, using the same stimulation method (Fig. 7B upper panels). This result indicates that CAG_200_ mice have a muscle defect associated with energy metabolism.

**Table 1:** Phrenic nerve-evoked muscle contracture force in isolated mouse diaphragm.

Line	Age (months)	Sex	Muscle contracture (g)
NT	6	F/M	0 (n = 3)
CAG_0_-10	6	F/M	0 (n = 3)
CAG_23_-16	2	F/M	0 (n = 3)
CAG_200_-62	5	M	0.62
	7	F	1.75
	7	M	1.25
	8	F	0.62
	9	F	0.38
	9	F	1.75
CAG_200_-32	6	M	1.02
	10	F	0.38
	10	F	0.93

NT, non-transgenic; F, female; M, male; n, number of mice analyzed.

All transgenic mice used in this assay were heterozygous.

### RNA foci and splicing analysis

**Figure 8:**
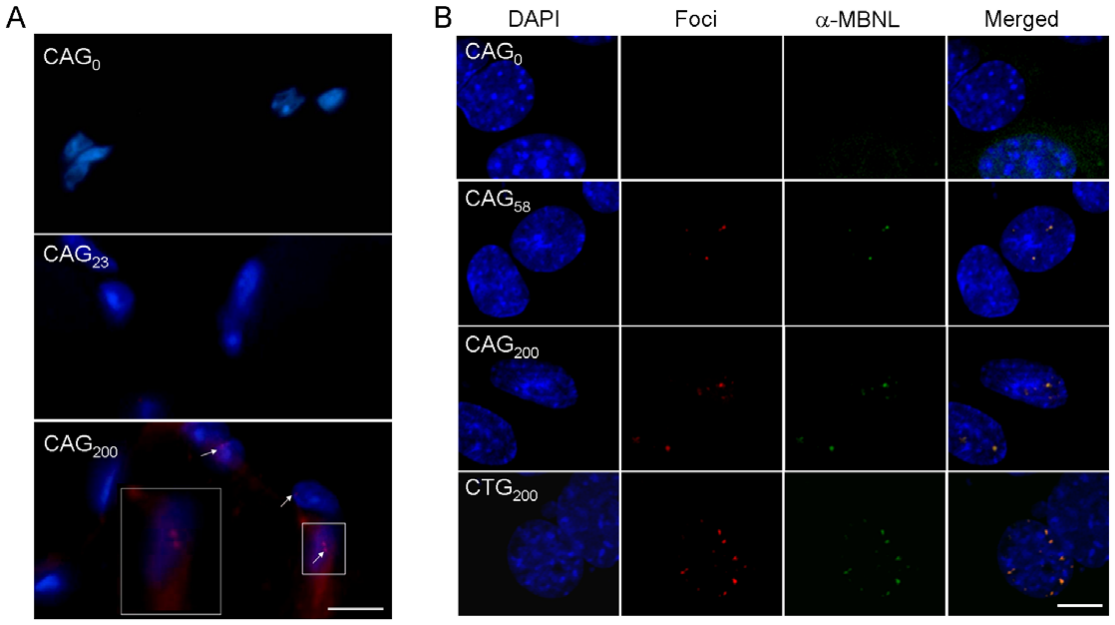
Fluorescence *in situ* hybridization detection of nuclear foci formation. (A) Frozen muscle sections from CAG_0_, CAG_23_ and CAG_200_ mice were hybridized with a Cy3-(CTG)_13_ probe (red). The nuclei were counterstained with DAPI (blue). Merged images of red and blue signals are shown. Nuclear foci (arrows) are only detected in CAG_200_ sections. (B) C2C12 cells transfected with pEGFP-CAG_0_/_58_/_200_ and pEGFP-CTG_200_ were hybridized with Cy3-(CTG)_13_ and Cy3-(CAG)_13_ probes, respectively. Distribution of endogenous MBNL proteins was visualized by immunostaining with an anti-MBNL antibody and a FITC-conjugated secondary antibody (green). Merged images showing superimposition of red and green signals (yellow) demonstrate that MBNL proteins are colocalized with expanded CAG and CUG repeats. Scale bars, 10 µm.

Expression of transcripts with expanded CUG or CAG repeats has been shown to result in nuclear accumulation of discrete RNA foci [20], [38], [41], [42]. Similar inclusions are also found in cells expressing Fragile X premutation CGG repeats [32]. To determine if expression of untranslated CAG repeats in muscle also induce RNA foci formation, we performed fluorescence *in situ* hybridization (FISH) on muscle sections from different lines of mice (Fig. 8A) and on C2C12 myoblasts that were transfected with untranslated CAG or CUG repeats (Fig. 8B). Punctuate nuclear foci were detected by a labeled (CTG)_13_ probe in CAG_200_ cells, but not by a (CAG)_13_ probe (data not shown). As expected, RNA foci were also detected in CUG_200_-expressing cells, but not in cells expressing the CAG_0_ construct. The RNA foci were also found in myoblasts expressing 58 CAG repeats (CAG_58_). The proportion of cells with RNA foci was comparable among CAG_58_, CAG_200_ and CUG_200_-expressing cells, but the number of foci per nucleus was significantly increased in CAG_200_/CUG_200_-expressing cells (Table S3). Moreover, the foci observed in myoblast cells were colocalized with endogenous MBNL (Fig. 8B).

Next, we examined whether expression of untranslated CAG repeat expansions affected splicing, as has been observed with CUG repeat RNA. We performed RT-PCR for *cardiac troponin T* (*cTNT*), *chloride channel protein 1* (*ClC-1*), and *Mbnl1* genes using RNA isolated from cardiac and skeletal muscles of NT, CAG_0_ and CAG_200_ mice. There were no noticeable differences in the splicing patterns of these endogenous genes in the transgenic lines (Fig. S1). Thus, these results suggest that CAG repeat RNA may cause deleterious effects through pathways that are distinct from those utilized by CUG repeat RNA.

## Discussion

Previous data showing that CAG repeat RNA forms stable hairpin structures and nuclear foci both *in vitro* and *in vivo* have supported a role for CAG repeat RNA in pathogenesis. Recently, CAG repeat RNA was shown to induce toxicity in *Drosophila* and *C. elegans* [41], [42]. In this study, we provide the first experimental demonstration that non-coding CAG expansion can also be deleterious in a mammalian system.

In the tissues of CAG_200_ mice, the level of translated EGFP protein was significantly reduced, whereas the transcript level was not affected by the repeat sequence (Fig. 2B–D). Th effect on translation reduction has also been reported for non-coding CUG [48], [49], CGG [50] and CAG [42] repeats. One possible explanation for this effect is that the double stranded hairpin structures formed by these repeats [17], [36] lead to a common mechanism of translation impediment. Alternatively, the reduced protein level may result from nuclear retention of long repeat-containing transcripts. Accordingly, nuclear foci were observed in the muscle cells of CAG_200_ mice (Fig. 8), and transcripts containing expanded CUG, CAG and CGG repeats have all been shown to accumulate in the nuclei [19], [23], [32], [38], [42]. We did not detect any cryptic translation products containing expanded polyglutamine (Fig. 2E), which argues against a protein mechanism contributing to the observed phenotypes. The fact that CAG_0_ and CAG_23_ mice, which expressed high levels of EGFP protein, lacked a discernable phenotype further supports the notion that expanded CAG repeat RNA is deleterious.

Different lines of CAG_200_ mice displayed consistent myopathic changes such as internalized nuclei, fiber splitting, increased nucleus-to-fiber ratio and loss of fiber type distinction. These pathological features somewhat resemble those observed in the skeletal muscle of DM1 mice, which expressed expanded CUG transcripts [13], [14]. Other histological features such as the ragged blue fibers and the moth-eaten fibers, both typical of mitochondrial myopathies, have also been noted in muscle biopsies from DM patients [51]. However, the key features of DM, muscle wasting and myotonia, were not detected in CAG_200_ mice. In addition to their muscle phenotype, CAG_200_ mice showed a reduced breeding efficiency. Because the testis histology and sperm counts were normal in these mice, the reduced breeding efficiency could be attributable to the poor sperm motility and defective axoneme structures observed in the CAG_200_ transgenics (Fig. 6). Together, the features of the CAG_200_ phenotype are suggestive of mitochondrial dysfunction.

There is an increasing amount of evidence that mitochondrial dysfunction plays a role in the pathogenesis of neurodegenerative disorders [52]. For example, defects in energy metabolism have been demonstrated in the skeletal muscle from patients with HD [53], [54]. This observation is of particular interest because the defect is correlated with the expansion of CAG repeats. CAG_200_ mice displayed lower grip strength and reduced locomotor activity, indicating that these mice may have muscle weakness and altered energy metabolism. Consistent with this notion, significant contracture was measured in the diaphragm of CAG_200_ mice in a condition that normally does not trigger it (Fig. 7B and Table 1). Thus, some unknown factors associated with energy metabolism and/or mitochondrial function may be affected by CAG repeats. Although mitochondrial abnormalities have been previously reported in some DM patients [51], [55], it is noteworthy that myotonic discharge induced by non-coding CUG expansion was not detected in the CAG_200_ mice. Therefore, it appears that CAG RNA expansion does not trigger the same pathogenic effects as CUG expansion in the mammalian system (see below).

Several studies have shown that long CAG repeats form nuclear RNA foci similar to those formed by CUG repeats [38], [41], [42]. While in some cases the muscleblind protein was recruited to the foci [38], [42], splicing of the reporter constructs with pre-mRNAs that were misregulated in DM cells was not altered [38], [41]. We have consistently observed nuclear foci formation and MBNL colocalization in muscle cells expressing 200 CAG repeats (Fig. 8). In addition, the splicing patterns of *cTNT*, *ClC-1* and *Mbnl1* were not altered in CAG_200_ mice (Fig. S1). These results suggest that the pathways downstream of CUG and CAG repeats are separable. However, using RNA isolated from cultured myoblasts expressing CAG_200_ or CUG_200_ transcripts, we observed that splicing of the ryanodine receptor 1 (*Ryr1*), which is altered in DM1 [56], was shifted from an adult form to a neonatal form when the cells were induced to differentiate (unpublished data). In worms, expression of long CAG or long CUG repeat RNA caused a reduction in *vinculin* gene expression [42]. This effect was also observed in the *muscleblind* knockdown worms, suggesting that muscleblind may participate in both CAG and CUG repeat-induced toxicity. Interestingly, reduction of *vinculin* expression was also observed in CAG_200_-expressing mouse myoblasts (unpublished data). Thus, it cannot be excluded that CAG repeat RNA may act, at least in part, through common factors that are also affected by CUG repeat RNA.

In contrast to our study, is has also been reported that CAG repeat RNA is not toxic [39], [57], [58]. It should be noted, however, that the expanded alleles used in these studies ranged from 79 to 93 repeats, much shorter than the 200 repeats we used in this study. It has been demonstrated that the toxicity induced by CAG repeat RNA is dependent on length, with a size threshold of approximately 100 repeats [41], [42]. Therefore, the size of the CAG repeat RNA is critical for its pathogenicity. The discrepancy between study results may also be due to the presence or absence of cell type-specific factors that are important for mediating the observed effects. For example, CAG repeat RNA that caused neuronal dysfunction and degeneration in *Drosophila* did not affect external eye morphology [41]. Further investigation of CAG repeat RNA that is directed to different tissues should clarify whether there is a selective sensitivity to the deleterious effects of CAG repeat RNA in distinct cell types.

HD has long been viewed as a polyglutamine disease due to a protein toxicity. Recently, a double-stranded RNA binding protein, PKR, was shown to be active in HD-affected tissues and to preferentially bind to a mutant Huntingtin transcript [59], [60]. Since PKR also binds to expanded CUG repeat RNA and has been implicated in DM pathogenesis [61], it is possible that an RNA-mediated mechanism is also involved in HD. In this regard, it is noteworthy that CAG_200_ mice displayed an energy metabolism defect that is shared by HD. Interestingly, evidence demonstrating the presence of anti-sense CAG repeat transcripts in DM1 fibroblast cells has also been reported [62]. Thus, a complex mechanism that involves CAG repeat RNA in the pathogenesis of DM1 cannot be excluded, and, moreover, it is supported by the observation that the phenotypes of CAG_200_ mice and DM1 mice are partially overlapping. In this study, pathologic findings in multiple transgenic lines clearly demonstrated that transcripts with expanded CAG repeats are deleterious in mice. Our data, together with previous findings, suggest that an RNA mechanism may participate in human diseases with long CAG repeats.

## Materials and Methods

### Transgene construction

A 1.5 kb 5′-flanking sequence of mouse gamma-sarcoglycan (gsg) gene was excised by KpnI and XmaI from plasmid pGSG-EGFP-1 (kindly provided by Dr. Noguchi) [43], and inserted at the same sites upstream of the EGFP sequence in vector pEGFP-1 (Clonetech). This vector was used as a backbone for insertion of CAG repeat sequence. The CAG repeat sequence was generated by polymerase chain reaction (PCR) using two complementary primers, (CTG)_10_ and (CAG)_17_, which were denatured at 94°C for 5 min, and then amplified by 30 cycles of 94°C, 30 sec; 37°C, 1 min; 72°C, 6 min, and a final extension at 72°C for 10 min. The PCR products were separated on a gel and the appropriate sized fragments were recovered and ligated to pGEM-T Easy vector (Promega). The number and integrity of CAG repeats in each clone was then determined by sequencing. Two fragments containing 23 and 200 CAG repeats were cut out by EcoRI at the multiple cloning site of pGEM-T Easy, and inserted at a modified NotI site which is downstream of the EGFP's stop codon and upstream of the SV40 polyadenylation sequence in the pGSG-EGFP vector. These constructs were mapped and sequenced to confirm the correct position, orientation, integrity and number of CAG repeats.

### Production of transgenic mice

The transgene fragments containing a 1.3-kb 5′-flanking sequence of the GSG gene (Fig. 1A) were cut out from vectors with *Afl*II. Transgenic mice were generated by microinjecting the purified transgene fragments into the male pronuclei of one-cell FVB/N embryo. Mice were genotyped initially by PCR using tail DNA with primers f2 (5′-CCACATGAAGCAGCACGAC-3′) and r1 (5′-GCTTTACTTGTACAGCTCGTC-3′) (Fig. 1A). PCR results were further confirmed by Southern blotting using 10 µg of tail DNA, digested with BamHI and hybridized with either an *EGFP*- or *GSG* promoter-specific probe. To confirm the length of CAG repeat sequence in mice, PCR-based Southern blotting was carried out by primers f1 (5′-CTGAAGTTCATCTGCACCAC-3′) and r2 (5′-CTACAAATGTGGTATGGCTG-3′) (Fig. 1A), using a labeled (CAG)_10_ probe as described previously [63].

### RT-PCR and Northern blotting

Total RNA was isolated from various tissues using the TRIREAGENT (Molecular Research Center Inc) according to manufacturer's protocol. Routinely, 5 µg of RNA was reverse transcribed with SuperScript II (Invitrogen) and one twentieth of the cDNA was used for PCR amplification using primers f2 and r1 as described above. Amplification of *GAPDH* (nt 458–774, accession no. NM_008084) was used as an internal control. For Northern blotting, 20 µg of total RNA from muscle was fractionated on a 1% agarose gel containing 3.7% formaldehyde, transferred to a nylon membrane (NEN), and hybridized with a ^32^P- labeled *EGFP* probe for 16 hr in 6XSSC, 2XDenhard's, 0.1% SDS, and 100 µg/ml denatured salmon sperm DNA, at 60°C. The membrane was then washed and exposed to IMAGING PLATE and read by a Phospho Image Analyzer (Fujifilm).

### Western Blot

Fresh tissues were homogenized in protein lysis buffer (15 mM Tris, 250 mM sucrose, 1 mM EDTA and 2 mM PMSF) and sonicated for 20 min. After centrifugation, about 100 µg of supernatants, as determined by the Bradford protein assay (Bio-Rad), were fractionated by 12.5% SDS-PAGE. The proteins were transferred to PolyScreen PVDF membranes (NEN), which were then blocked in 3% skim milk in Tris-buffered saline (TBS). The membranes were incubated in blocking solution with anti-EGFP (Living Colors A.v. peptide Antibody, 1∶500 dilution, BD Biosciences Clontech), anti-polyglutamine (1C2, 1∶2000 dilution, Chemicon) or anti-tubulin β(1∶1000 dilution, MD Bio) overnight, followed by washing in TBS and incubation with HRP-conjugated secondary antibodies (1∶3000 dilution, Santa Cruz) in 3% skim milk/TBS for 1 hr. After a final wash, bound antibodies were visualized by SuperSignal West Pico chemiluminescent substrate kit (Pierce). For polyglutamine-containing protein detection, two stably transfected cell lines expressing expanded ataxin-3 proteins with 54 and 109 CAG repeats (a gift from Dr. Hsieh M.) were used as positive controls.

### Histology and histochemistry

Muscle tissues of soleus and diaphragm were fixed in 4% paraformaldehyde/PBS overnight at 4°C, washed in PBS, dehydrated in increasing concentrations of ethanol and embedded in paraffin. Sections of 5 µm were cut and stained with hematoxylin and eosin. For histochemical reactions, fresh soleus muscle tissues were coated with OCT and frozen at -80°C. Cryostat sections of 8 µm were cut and immediately soaked in incubation medium. The succinate dehydrogenase, NADH-tetrazolium reductase and ATPase enzyme histochemistry were carried out as described by Cash and Blumbergs [64].

### Fluorescence *in situ* hybridization (FISH) and immunofluorescence staining

Cryosections (10 µm) of muscle tissue from adult mice and cultured C2C12 cells were used for FISH analysis using Cy3-labeled (CTG)_13_ and (CAG)_13_ probes. C2C12 cells were maintained in Dulbecco's modified Eagle's medium (DMEM, Gibco), supplemented with 4 mM L-glutamine, 1% penicillin/streptomycin, and 10% FBS at 37°C in 5% CO_2_. Cells (3×10^5^) were plated into 35 mm dishes containing pre-sterilized coverslips for 24 hours, and transfected with 2 µg of plasmids (pEGFP-CAG0/58/200 and pEGFP-CTG200, where repeat sequences are inserted in the 3′-UTR of *EGFP*) using Lipofectamine™2000 (Invitrogen) according to the manufacturer's instruction. FISH analyses were carried out primarily as described [42] except that cells were fixed in 4% paraformaldehyde (PFA) for 30 min and were permeabilized with 0.2%Triton X-100 (in PBS/DEPC) for 5 min before post-fixation in 2% PFA. After post-hybridization washes, cells were blocked in 1× casein solution (Vector) in TBST (0.05 M Tris-HCl, 0.15 M NaCl, 0.1% Tween-20, pH 7.6) for 1 hour at room temperature and then incubated with an anti- MBNL monoclonal antibody (Santa Cruz, 1∶200 dilution) at 4°C overnight. Afterwards, slides were washed three times in TBST and incubated with a FITC-conjugated goat anti-mouse IgG (Santa Cruz, 1∶500 dilution) for 2 hours at room temperature. After final washes in TBST, the slides were stained with DAPI and mounted in fluorescence mounting medium (DAKO).

### Phenotype analysis

A total of 60 mice at 2, 4 and 6 months of age were analyzed. Each 15 mice of heterozygous CAG_200_, CAG_23_, CAG_0_ and nontransgenics were divided into three age-matched groups. For grip strength test, the mice were placed with their forelimbs on a narrow bar and the amount of time taken for the mouse to fall was assessed. For cage activity test, mice were placed on to a grid of squares (3×3 cm) in a cage, and were allowed 5 min to settle before testing began. The mouse was observed for 3 min and the number of squares crossed by the mouse per minute was recorded. Both tests were repeated three times with at least 10 min between each test.

### Sperm counts, Rh123 staining, and electron microscopy

Male mice at 3 to 4 months of age were sacrificed and the cauda epididymides were excised and placed in 4 ml of phosphate-buffered saline (PBS). Following incubation at 37°C for 1 hour, the spermatozoa were observed and counted by a hemacytometer under a light microscope. Sperm from 6 males of each group was counted and analyzed, and the numbers were averaged. For Rh123 staining, the spermatozoa were incubated with Rh123 (5 µg/ml in PBS) at 21°C for 30 min, submitted to a centrifugation step (500×*g* 5 min) and then incubated with fresh PBS at 21°C for 45 min to eliminate nonspecific binding of the dye. After recentrifugation at 500×*g* for 5 min, the spermatozoa were counterstained with PI (0.05 µg/ml in PBS) for 5 min and then subjected to flow cytometric analysis. For electron microscopy, the spermatozoa released from epididymides were washed with PBS for three times, pelleted by centrifugation at 1000×*g* for 20 min, and fixed in 4% paraformaldehyde plus 2.5% glutaraldehyde in 0.1 M cacodylate. Following post fix in 2% osmium tetroxide, the pellets were embedded in spurr. The thin sections were examined in a Joel 1200EX electron microscope after double staining with uranyl acetate and lead citrate.

### Muscle action potential and contracture recordings

Conventional microelectrode recording techniques were used. The glass microelectrodes were filled with 3 M KCl and had resistance in the range 5–15 MΩ. The mouse diaphragm was placed in modified Krebs solution (130.6 mM NaCl, 4.8 mM KCl, 1.2 mM MgSO_4_, 12.5 mM NaHCO_3_, 2.5 mM CaCl_2_, 10 mM glucose, pH 7.2–7.4) and gased with 95%+5% CO_2_. Muscle fiber action potentials were elicited by intracellularly injected depolarizing current (12 nA, 100 ms), the pulses being delivered through the bridge mode of the Axoclamp-2B (Axon Instruments). The waveforms were recorded and analysis on a computer with P-Clamp 9.0 software (Axon Instruments). For contracture recording, the phrenic nerve-hemidiaphragm was isolated as previously described [65]. The diaphragm was suspended in 10 ml Krebs solution at 37.0±0.5°C and constantly gassed with 95% O_2_+5% CO_2_. Subsequently the solution was substituted by either fresh Krebs solution or glucose-free Krebs solution (glucose substituted by equal moles of NaCl). The twitches of diaphragm were elicited by indirect stimulation of the phrenic nerve with supramaximal rectangular pulses of 0.05 ms duration at 5 Hz, and recorded with an isometric transducer (Grass FT.03) on a Gould Model TA240 polygraph. The muscle resting tension was adjusted to 2 g, and the preparation was then allowed to balance for 15–20 min with 0.1 Hz before starting the 5 Hz stimulation experiment.

### Ethics Statement

This study was conducted according to the guidelines recommended by the National Laboratory Animal Center. The animal use protocol was approved by the Institutional Animal Care and Use Committee of Chung-Shan Medical University (Permit Number: 207, 315).

## Supporting Information

Figure S1 RT-PCR analysis of *cTNT*, *ClC1*, and *Mbnl1* alternative splicing. RNA isolated from cardiac and skeletal muscle cells of neonatal and adult non-transgenic (NT), CAG_0_, CAG_200_ mice was subject to RT-PCR analysis. Forward and reverse primers used for *cTNT*, *ClC1*, and *Mbnl1* were located on exon 2/8, exon 5/8, and exon 4/6, respectively. Twenty five cycles of PCR reactions were carried out and ^32^P-labeled products were resolved on 5% polyacrylamide gels. Major splicing variants with exon inclusion/exclusion are indicated. No difference in splicing patterns of these genes is found among either neonatal or adult NT, CAG_0_, CAG_200_ mice.
(TIF)

Table S1 Transgenic lines used for experimental analysis.
(DOC)

Table S2 Quantification of structural defects in sperm.
(DOC)

Table S3 Quantification of RNA foci formation.
(DOC)
