# Supplementary figures and images for: Long Tract of Untranslated CAG Repeats Is Deleterious in Transgenic Mice
Source: PLoS One. 2011 Jan 21;6(1):e16417. doi: 10.1371/journal.pone.0016417 (PMC3025035; doi:10.1371/journal.pone.0016417)

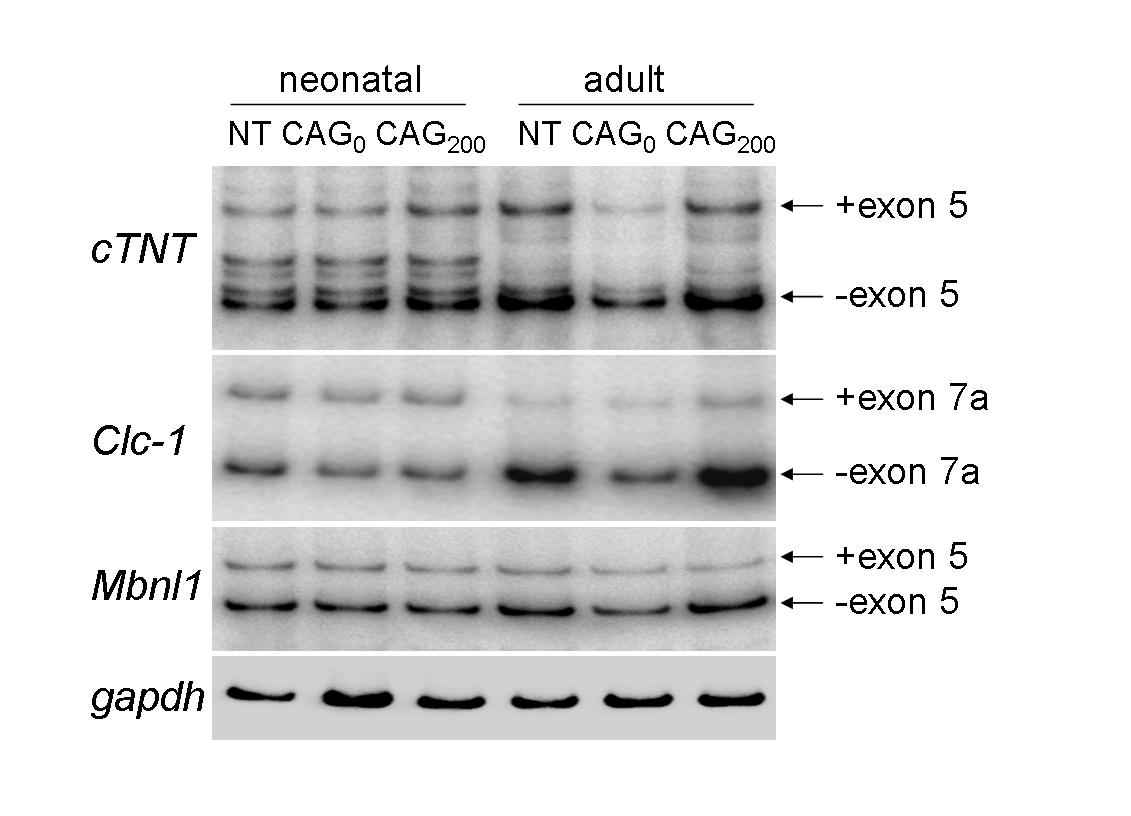

Supplement: Figure S1 — RT-PCR analysis of cTNT, ClC1, and Mbnl1 alternative splicing. RNA isolated from cardiac and skeletal muscle cells of neonatal and adult non-transgenic (NT), CAG0, CAG200 mice was subject to RT-PCR analysis. Forward and reverse primers used for cTNT, ClC1, and Mbnl1 were located on exon 2/8, exon 5/8, and exon 4/6, respectively. Twenty five cycles of PCR reactions were carried out and 32P-labeled products were resolved on 5% polyacrylamide gels. Major splicing variants with exon inclusion/exclusion are indicated. No difference in splicing patterns of these genes is found among either neonatal or adult NT, CAG0, CAG200 mice. (TIF) [file pone.0016417.s001.tif]
