# Supplementary material for: Long Tract of Untranslated CAG Repeats Is Deleterious in Transgenic Mice
Source: PLoS One. 2011 Jan 21;6(1):e16417. doi: 10.1371/journal.pone.0016417 (PMC3025035; doi:10.1371/journal.pone.0016417)
Supplement: Table S1 — Transgenic lines used for experimental analysis. (DOC) [file pone.0016417.s002.doc]

**SUPPLEMENTARY TABLE**

Table S1. Transgenic lines used for experimental analysis.

| Transgenic | CAG_0_ | | | CAG_23_ | | | | | CAG_200_ | | | |
| --- | --- | --- | --- | --- | --- | --- | --- | --- | --- | --- | --- | --- |
| Lines | 10 | 24 | 41 | 11 | 25 | 16 | 31 | 35 | | 32 | 57 | 62 |
| Expression |  |  |  |  |  |  |  |  | |  |  |  |
| RT-PCR | + |  | + |  |  | + | + | + | | + | + | + |
| Northern | + |  | + |  |  |  | + |  | | + | + | + |
| Western | + |  | + | + | + | + | + |  | | + | + |  |
| Fluorescent | + |  |  |  | + |  |  |  | | + |  |  |
| H&E, IHC | + | + |  | + |  | + | + | + | | + |  | + |
| Foci | + |  |  |  |  | + |  |  | | + |  | + |
| Sperm assay | + |  | + | + |  | + | + |  | | + | + | + |
| Behavioral assay |  | + |  | + |  |  |  |  | |  |  | + |
| Electrophysiology | + |  |  |  |  | + |  |  | | + |  | + |
