# Supplementary material for: Long Tract of Untranslated CAG Repeats Is Deleterious in Transgenic Mice
Source: PLoS One. 2011 Jan 21;6(1):e16417. doi: 10.1371/journal.pone.0016417 (PMC3025035; doi:10.1371/journal.pone.0016417)
Supplement: Table S2 — Quantification of structural defects in sperm. (DOC) [file pone.0016417.s003.doc]

**SUPPLEMENTARY TABLE**

Table S2. Quantification of structural defects in sperm^a^.

| Transgenic line | CAG_0_  10 | CAG_23_  16 | CAG_200_  62 32 57 | | |
| --- | --- | --- | --- | --- | --- |
| Number of sperm inspected | 9 | 9 | 8 | 11 | 7 |
| Number of sperm with axoneme defects | 0 | 0 | 5 | 8 | 4 |

^a^Structural defects were observed with an electron microscope. Two fields were viewed for each mouse.
