# Supplementary material for: Long Tract of Untranslated CAG Repeats Is Deleterious in Transgenic Mice
Source: PLoS One. 2011 Jan 21;6(1):e16417. doi: 10.1371/journal.pone.0016417 (PMC3025035; doi:10.1371/journal.pone.0016417)
Supplement: Table S3 — Quantification of RNA foci formation. (DOC) [file pone.0016417.s004.doc]

**SUPPLEMENTARY TABLE**

Table S3. Quantification of RNA foci formation.

|  | CAG_0_ | CAG_58_ | CAG_200_ | CUG_200_ |
| --- | --- | --- | --- | --- |
| Nuclei with foci^a^ | 0% | 13.6% | 17.4% | 13.1% |
| Foci/nucleus | 0 | 3.4 (n=14) | 6.7* (n=20) | 6.5* (n=16) |

^a^Ten microscopic fields were counted for each CAG/CUG expressing cell line. Percentages were determined by dividing the number of nuclei with foci by the total number of nuclei.

*, significantly different from CAG_58_, *P*<0.01.
